# Follicular dendritic cell sarcoma arising in the stomach and abdominal cavity: A case report

**DOI:** 10.1097/MD.0000000000034289

**Published:** 2023-08-04

**Authors:** Ting Zhan, Shanshan Xing, Chunhua Lu

**Affiliations:** a Department of Radiology, the Second Affiliated Hospital of Nanchang University, Nanchang, China.

**Keywords:** follicular dendritic cell sarcoma, imaging features, stomach and abdominal cavity

## Abstract

Follicular dendritic cell sarcoma (FDCS) is a rare mesenchymal tumor that typically develops in lymph nodes; it is clinically uncommon and has only occasionally been documented in cases of soft tissue, liver and spleen, and retroperitoneum; it is also extremely uncommon to develop in the stomach.

**Patient concerns:**

A 64-year-old woman who discovered a lump in her left upper abdomen 6 months prior and was taken to the hospital due to excruciating abdominal pain.

**Diagnosis:**

An abdominal computed tomography scan showed a soft tissue mass around the cardia. The immunohistochemical and postoperative histopathology results were compatible with FDCS.

**Interventions:**

The patient underwent “radical total gastrectomy and esophagojejunostomy” (Roux-Y anastomosis).

**Outcomes:**

The patient recovered well 2 months after surgery.

**Lessons:**

We report a case of FDCS occurring in the stomach and abdominal cavity, which was unique in terms of clinical location, clinical presentation, and imaging signs. This case report aims to enhance clinicians’ understanding and diagnosis of FDCS in the stomach and abdominal cavity and reduce the rate of clinical misdiagnosis.

## 1. Introduction

Approximately 0.4% of all soft tissue sarcomas are follicular dendritic cell sarcoma (FDCS), an uncommon intermediate malignancy.^[1]^ In 1986, Monda et al described and discussed the first case of FDCS.^[2]^ FDCS often occurs in lymph nodes, most commonly in the neck, mediastinal, and axillary lymph nodes. The tumors grow slowly and have no obvious symptoms, which can lead to recurrence and distant metastasis.^[3]^ Clinically, FDCS at extranodal sites is uncommon, with sporadic reports of occurrences in soft tissue, the liver and spleen, the gastrointestinal tract, and the retroperitoneum.^[4]^ FDCS occurring in the stomach is extremely rare. No significant gender difference has been reported in young adult patients with FDCS.^[5]^ Because the clinical and imaging signs of FDCS lack specificity, physical examination plus imaging cannot distinguish the difference between FDCS and other disorders. As a result, it has been difficult for clinicians to fully understand FDCS, which makes FDCS diagnosis challenging.^[6,7]^

In this report, we present the imaging findings of a case of FDCS occurring in the stomach and abdominal cavities. We present the following case in accordance with the case report guidelince reporting checklist.

## 2. Case presentation

The patient was a 64-year-old female. Six months prior to admission, she discovered a firm, immobile mass in her left upper abdomen. Once the patient was admitted to the hospital for treatment of a gradually increasing mass, the mass was approximately the size of a fist. When touched the patient was accompanied by severe, persistent, and dull pain in the abdomen and associated with black stools. The patient had a history of hypertension for more than 10 years and had been taking amlodipine besylate regularly for blood pressure control. Physical examination of the patient revealed no obvious abnormalities, and no obvious lymph node enlargement was observed on the body’s surface. Laboratory test results showed ESR103 mm/h (normal range: 0–20 mm/h), alkaline phosphatase 148.93 U/L (normal range: 50–135 U/L), total bile acid 19.71 μmol/L (normal range: 0–10 μmol/L), total cholesterol 5.28 mmol/L (normal: <5.18 mmol/L), tumor supplied group of factors 89.18 U/mL (normal range: 0–64 U/mL), and fibrin concentration of 5.29 g/L (normal range: 0–24 g/L). The hepatitis B surface antibody and hepatitis B surface core antibody were both positive. No abnormalities were observed in gastric cancer markers.

**Figure 1.**
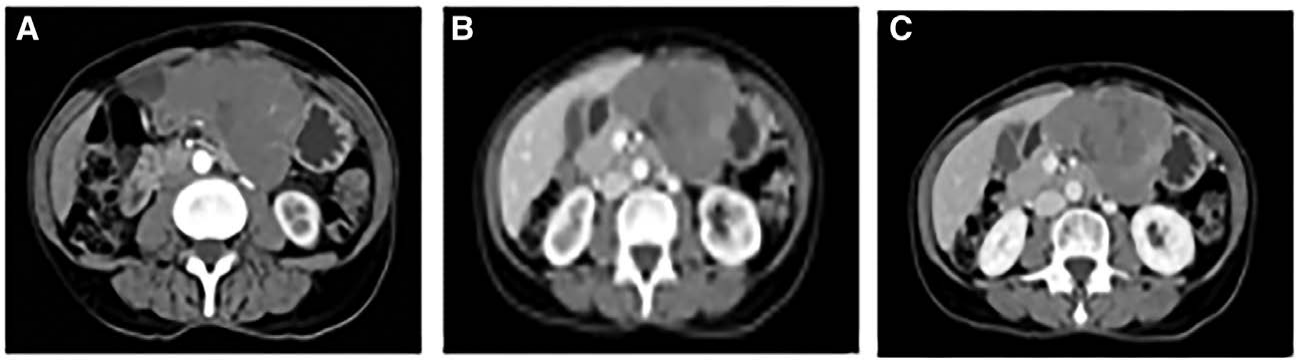
Computed tomography scans of the tumor. (A) Arteria phase. (B) Portal venous phase. (C) Delayed phase.

A full abdominal computed tomography (CT) scan showed an irregularly shaped soft tissue mass with a maximum cross-sectional area of approximately 90 mm × 60 mm in the lesser curvature of the stomach, with unclear boundaries, uneven density within the mass, and patchy irregular low-density necrotic areas were seen. The lesion was mildly intensified, and the core necrotic area was not enhanced in the arterial phase of the enhanced scan. The lesion was found to be supplied by the left gastric artery. The lesion’s degree of enhancement increased during the portal venous phase and increased more during the delayed phase, resulting in “progressive enhancement” overall (Fig. 1). The adjacent tissue structures were compressed, and multiple soft tissue nodules of variable size were seen in the greater omentum area. Our clinical diagnosis was “gastrointestinal stromal tumor,” and surgical treatment was performed. A hard diffuse mass was observed in the body of the stomach, approximately 5 cm from the cardia. The mass had penetrated the plasma membrane and had a nodular surface. The perigastric lymph nodes of Groups 3, 7, 8, and 9 were enlarged, hard, and fused into a mass, and a multicenter mass of soy size was seen in the greater omentum. The intraoperative diagnosis was a “malignant tumor of the stomach,” and “radical total gastrectomy + esophagojejunostomy” (Roux-Y anastomosis) was performed. The patient demonstrated good recovery 2 months after the surgery.

**Figure 2.**
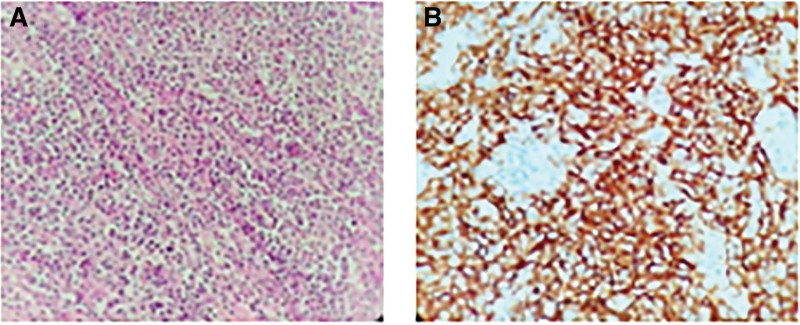
Tumor histology. (A) The section demonstrates fusiform cells in whirling patterns (hematoxylin and eosin stain; 400× original magnification). (B) Positive staining for CD21 is a characteristic feature of follicular dendritic cell sarcoma (400× magnification).

Histopathological examination of the resected surgical specimen showed diffuse infiltrative growth of tumor cells from the submucosa to the subserosa of the stomach and invasion of the peritoneum. The tumor cells were spindle or spindle-shaped, round, or ovoid in bundle, swirl, and woven distribution. Cellular heterogeneity was evident in focal areas, with small lymphocytes scattered in the interstitium. Immunohistochemistry showed that the tumor cells were positive for CD21, CD23, CD35, CD68, CD163, vimentin, and anti-smooth muscle antibody, focally positive for S-100 protein, partially positive for CD45, and involved approximately 40% of Ki-67 proliferation index hotspots (Fig. 2). cytokine, CD117, DOG1, HMB-45, anaplastic lymphoma kinase, and CD1α were negative, but most of the reactive lymphocytes were positive for CD2, CD3, CD5, and CD7. A few scattered lymphocytes were positive for CD20 and PAX-5, and CD34 positively marked many proliferating vessels. The results were detected by in situ hybridization and showed that EBER was negative. Based on a combination of morphological and immunohistochemical features, this case was diagnosed as an FDCS stomach and abdominal cavity tumor.

## 3. Discussion

FDCS, a moderate sarcoma that develops from follicular dendritic cells, has an estimated risk of metastasis and local recurrence rate in the 40%–50% range.^[8,9]^ Approximately one-third of all cases of FDCS are found in the cervical and axillary lymph nodes.^[10]^ However, extranodal FDCS can virtually affect any organ in the body, including the tonsils, thyroid, adrenal glands, nasopharynx, lungs, gastrointestinal tract, liver, pancreas, spleen, and intracranium.^[11,12]^ FDCS can occur in many locations throughout the body and can appear as different tumors or even inflammatory diseases.^[13]^ The clinical manifestations of FDCS are often atypical and often have a chronic course. FDCS in the lymph nodes usually presents as a slow-growing, painless mass,^[8]^ while FDCS occurring in the abdominal cavity may be associated with abdominal pain, abdominal distention, and abdominal masses.^[7]^ In some cases, FDCS is also associated with systemic symptoms such as fever, weight loss, fatigue, and night sweats.^[9]^ Typically, gastrointestinal FDCS tumors begin as slow-growing, painless masses.^[14]^ However, these tumors are difficult to detect and accurately diagnose by clinical examination alone because of the nonspecific clinical presentation of FDCS.

The diagnosis of FDCS is mainly based on histopathological and immunohistochemical examination. The tumor cells in the current case report showed an oval or spindle shape with a widespread, nodular distribution. This is consistent with previous related reports in that the cytological features are characterized by spindle-to-ovoid or fusiform cell-forming fascicles and whole, diffuse sheet or nodular shapes with lymphocyte infiltration.^[15]^ The follicular dendritic cell markers, CD21, CD23, and CD35, which are the primary markers for differentiation from other diseases, including dendritic sarcoma, soft tissue sarcoma, lymphoma, and especially interdigitating dendritic cell sarcoma, were found to be positive in this patient’s immunohistochemical analysis.^[16]^ Furthermore, a significant prognostic indicator for FDCS is positive Ki-67 expression.^[15]^ These immunohistochemical results provided important clues that helped to differentiate FDCS from non-FDCS disease and improve the accuracy of FCDS diagnosis.

The location of the FDCS in our patient was highly uncommon; the liver and retroperitoneum have been reported to be the most common primary sites for FDCS in the abdomen. The primary tumor in this case metastasized to the perigastric lymph nodes and was situated close to the gastric cardia. It invaded the abdominal cavity and formed several tumor nodules of various sizes. Similar to a previous study that reported FDCS, CT revealed a soft tissue mass with blurred borders, heterogeneous density, and “progressive enhancement.” The density of the FDCS mass is usually uneven on CT scans, typically appearing as an isointense or slightly hypointense soft tissue shadow. Low-density cystic necrosis or massive calcification can be seen in some lesions, and uneven enhancement can be seen on dynamic enhancement CT.^[17]^ FDCS typically presents as single or multiple large soft tissue masses with heterogeneous density, central cystic degeneration, hemorrhage, and necrosis. CT multiphase enhancement scans show that the solid component of FDCS is typically mildly enhanced, with progressive enhancement overall. Consistent with previous reports in the literature,^[18]^ the pattern of enhancement and the presence of blood-supplying arteries around the tumor suggest that an intra-abdominal FDCS is a blood-rich tumor. In terms of imaging, Leipsic et al,^[19]^ who first documented the CT indications of mediastinal FDCS in 2007, reported a case of FDCS that showed a sizable soft tissue mass accompanied by central calcification on a regular CT scan. An instance of FDCS between the left lobe of the liver and the lesser curvature of the stomach was also reported by Long-Hua et al.^[20]^ Their patient’s enhancement scan revealed moderately uneven enhancement, several necrotic areas within the tumor, distinct tumor boundaries, and there was no evident retroperitoneal lymph node enlargement. These enhanced CT scan results were similar to the main imaging signs of FDCS summarized by Kang et al,^[21]^ including clear tumor boundaries accompanied by regional lymph node enlargement, uniform enhancement, internal necrosis, calcification, and other imaging signs.

Currently, there is no uniform standard of care for FDCS, and complete surgical resection is the primary treatment option for both primary and recurrent lesions.^[9]^ The effects of radiation and chemotherapy have been reported to be nonsignificant, and adjuvant chemotherapy has had no discernible impact on overall patient survival.^[22]^ A mostly inert course and modest malignancy characterize the somewhat unusual clinical course of FDCS. And its biological behavior and prognosis are related to patient age (approximately 40 years old), tumor size (>6 cm), lymphoplasmacytic infiltration, tumor cell nuclear division count (>5/10 high-powered field), lymph node metastasis, positive Ki-67, and massive coagulative necrosis.^[23]^

## 4. Conclusions

Intra-abdominal FDCS is very rare. The preoperative diagnosis is difficult because patient age of onset and clinical and imaging manifestations are nonspecific. However, certain CT imaging features can not only provide a basis for differential diagnosis but also guide surgery. We hope our report will provide clinicians with information that can be helpful in diagnosing this disease when encountering these types of tumors.

## Acknowledgments

The authors would like to thank the patient and her family for providing informed consent for publication.

## Author contributions

**Conceptualization:** Ting Zhan, Shanshan Xing.

**Data curation:** Ting Zhan, Shanshan Xing.

**Writing – original draft:** Ting Zhan, Shanshan Xing.

**Writing – review & editing:** Chunhua Lu.
